# Synthetic biology as a source of global health innovation

**DOI:** 10.1007/s11693-013-9117-3

**Published:** 2013-07-19

**Authors:** Jenny Rooke

**Affiliations:** San Francisco, CA USA

**Keywords:** Global health, Synthetic biology, Innovation, Funding

## Abstract

Synthetic biology has the potential to contribute breakthrough innovations to the pursuit of new global health solutions. Wishing to harness the emerging tools of synthetic biology for the goals of global health, in 2011 the Bill & Melinda Gates Foundation put out a call for grant applications to “Apply Synthetic Biology to Global Health Challenges” under its “Grand Challenges Explorations” program. A highly diverse pool of over 700 applications was received. Proposed applications of synthetic biology to global health needs included interventions such as therapeutics, vaccines, and diagnostics, as well as strategies for biomanufacturing, and the design of tools and platforms that could further global health research.

## Why apply synthetic biology to global health?

Technological advances in the field of health continually bring us closer to a world where a healthy life is a real option for every individual on the planet, regardless of geography, culture, or socioeconomic status. However, these benefits tend to accrue disproportionately to the developed world; the need is still great for solutions that can diagnose illness, protect against infection, and treat disease in a broad array of low-cost settings with developing-world healthcare systems and limited infrastructure. Innovative, technology-enabled alternatives to standard approaches for developing novel human health solutions are required to solve some of the most difficult global health challenges, where solutions must both comprise breakthrough science and be practical, affordable, and accessible to people in need. Synthetic biology (Andrianantoandro et al. 2006) is one such approach with the potential to deliver global health solutions, including vaccine and drug creation, diagnostics, and combinations of interventions within a single biological system.

Characterized by a bottoms-up approach to designing biological systems for a specific purpose, synthetic biology offers opportunities for achieving goals that observation and analysis do not. A synthetic goal forces science to broach and solve problems that are not readily encountered through analysis alone, driving the creation of new solutions. Like global health, synthetic biology is highly multidisciplinary, bringing together tools and perspectives from traditional health and life sciences disciplines such as cell biology, chemistry, genetics, pathology, and immunology, among others, as well as disciplines less tightly linked to the health sciences, such as engineering, materials science, and fabrication. And synthetic biology is already showing promise in fields such as industrial biology and biofuels for improving the economics and accessibility of products, for example by reducing manufacturing costs and improving production efficiency.^1^

For these reasons, synthetic biology has the potential to contribute novel and radical innovations to the pursuit of new global health solutions. Specific examples of how synthetic biology could be applied to global health needs might include:

- A novel intervention to prevent infectious disease using chemicals, materials or organisms engineered via synthetic biology approaches;
- Chemicals or materials biofabricated to improve the efficacy of disease treatment, or to increase the chemical diversity available for new drug discovery/development;
- A diagnostic biosensor for a global health indication using genetic circuitry or other approaches for stimulus and response induction;
- A synthetic instance of a biological system to accelerate development of global health interventions (e.g. a predictive model for preclinical drug or vaccine testing);
- A synthetic instance of a biological system to test and further our understanding of that system, addressing a critical knowledge gap in global health (e.g. a synthetic model of disease pathogenesis).

To be sure, synthetic biology faces significant hurdles on the road to delivering global health solutions. These include potential safety concerns; in particular, preventative interventions such as vaccines for global infectious diseases have a high safety bar. Delivery challenges inherent to an innovative idea (e.g., ensuring that an intervention reaches and enters the relevant tissues or cells in a patient) can become outright barriers when considered in a developing-world context, where infrastructure, technologies, or expertise can easily be a limiting factor. While synthetic biology has the potential to decrease costs of some products, biological interventions in general are not inherently low-cost, and may not be cost-effective in global health settings. In addition, synthetic biology is subject to the same ethical, legal, and social (ELSI) concerns that pertain to genetic engineering, sometimes in magnified form. ELSI issues are outside the scope of this article; see Anderson et al. (2012) for a recent framing of the issues and a perspective on addressing them, as well as work appearing in this Special Issue (Douglas and Stemerding 2013; Hollis 2013; Van den Belt 2012).

## Results of an open grant call

Wishing to harness the emerging tools of synthetic biology for the goals of global health, in 2011 the Bill & Melinda Gates Foundation put out a call for grant applications to “Apply Synthetic Biology to Global Health Challenges” under its “Grand Challenges Explorations” (GCE) program.^2^ The Gates Foundation’s Global Health division aims to harness advances in science and technology to save lives in developing countries. GCE grants afforded an ideal mechanism by which to direct the attention and talents of the nascent field of Synthetic Biology toward Global Health needs of which it might otherwise not be aware. In addition, it was intended to attract applicants from disciplines beyond a typical biomedical background, such as bioengineering and biophysics, thus increasing the cross-disciplinary participation and leading to more innovations in the synthetic biology approach to global health.

Over 700 applications were received across two rounds of the grant call. A wide array of global health needs was represented, with the majority of proposed projects targeting infectious diseases such as HIV, malaria and tuberculosis. Proposed applications included creating synthetic biology variations on traditional health interventions such as therapeutics, vaccines, and diagnostics, as well as strategies for biomanufacturing, and the design of tools and platforms that could further global health research. The applicant pool was highly diverse on dimensions of geography and institution, indicative of the breadth of interest and activity in the field of synthetic biology.

A panel of reviewers assessed applications on multiple dimensions including responsiveness to the grant call, fit with Gates Foundation priorities, innovativeness, and potential for ultimate impact, including practical implications such as cost and deliverability. A total of thirty $100,000 grants were awarded to fund projects seeking to apply synthetic biology to global health needs, summarized in Table 1. The resulting project portfolio reflects the global health priorities of the Bill & Melinda Gates Foundation,^3^ and thus cannot be considered to be generally representative of global health research, synthetic biology research, or the intersection thereof. Nevertheless, the sample set is sufficiently large and diverse to allow interesting themes to emerge. These are discussed below, grouped by application area.

**Table 1:** List of projects funded under the Grand Challenges Explorations Program, “Apply Synthetic Biology to Global Health Challenges”

Project title	Principal investigator(s)	Institution	Summary
*Diagnostics/biosensors*			
A household yeast biosensor for cholera	Virginia Cornish, Nili Ostrov	Columbia University	Engineer baker’s yeast to produce the red tomato pigment lycopene when exposed to the cholera pathogen in drinking water
Bacteriophage-based LAMP for pathogen detection	Héctor Morbidoni	Universidad Nacional de Rosario	Develop a biosensor to detect bacterial pathogens using modified bacteriophages and an isothermal DNA amplification process
Microbial biosensor for diagnosing leishmaniasis	Darren Zhu	Synbiosys, LLC	Engineer a bacterium with cell surface receptors that are activated and amplified by the presence of Leishmania proteases to produce a colorimetric readout that can rapidly diagnose leishmaniasis in field conditions
Multi-diagnostic platform derived from olfactory receptors	Sergio Botero	Rockefeller University	Build and test a library of yeast cells that express olfactory receptors encoded with a reporter gene that can react to various metabolic and infectious diseases, to be used in a diagnostics platform to detect multiple diseases at a time
Parasite protease biosensors	Paul Freemont	Imperial College London	Develop and test a self-replicating biosensor that can quickly detect proteases released by parasites
Pigment-based, low-cost, portable nutrition status tests	Mark Styczynski	Georgia Institute of Technology	Create portable, low-cost, bacteria-based genetic circuits to measure blood micronutrient levels without requiring sophisticated instrumentation to perform or read the test
Programmable genetic memory in bacteroides: diagnosis of diarrheal disease	Christopher Voigt, Michael Fischbach, Justin Sonnenburg	Massachusettes Institute of Technology, University of California San Francisco, Stanford	Engineer a strain of a common bacterial inhabitant of the human gut to contain genetic sensors that can report biomarkers for intestinal disorders in a stool sample
DNA nanodevice for pathogen detection	Eric Henderson	Iowa State University	Build an inexpensive and robust nanodevice that uses DNA as a scaffold to interact with proteins and nucleic acid markers of target pathogens. When this interaction occurs, the movement will be detected by a reader embedded in the device to create a visual readout of pathogen detection
Protein-based low-cost metabolite biosensors for pneumonia	Andriy Kovalenko, Nikolay Blinov, David Wishart	University of Alberta	Develop protein-based metabolite biosensors to create a simple, low-cost diagnostic test for pneumonia that is based on specific metabolite signatures found in urine
Nature-inspired nanoswitches for hiv antibodies detection	Francesco Ricci, Alexis Vallee-Belisle	University of Rome, Tor Vergata; University of California, Santa Barbara	Develop molecular nanoswitches that provide a visual cue when they bind to HIV antibodies for use in a rapid (1 min) diagnostic test to detect and quantify HIV antibodies in serum samples
Yeast receptors for a generic biomarker detection platform	Keith Tyo, Josh Leonard	Northwestern University	Engineer yeast-based biosensors that identify protein biomarkers in samples like blood and urine. An array of yeast strains could serve as a low-cost, in-home panel of diagnostics
*Diagnostic/biosensor plus therapeutic*			
A method to generate bacteriophages targeting enterobacteria	Mark van Raaij	Spanish National Research Council (CSIC)	Build a library of engineered bacteriophages that can recognize, infect, and kill a range of enterobacteria such as *Salmonella* and *E. coli*
Synthetic probiotic to identify and prevent cholera	James Collins, Ewen Cameron, Peter Belenky	Boston University, HHMI	Engineer the probiotic bacterium Lactobacillus gasseri to detect and kill Vibrio cholerae in the human intestine
*Therapeutics*			
Bacteriophage with programmable antibiotic activity	Feng Zhang	Broad Institute of MIT and Harvard	Engineer bacterial viruses to deliver enzymes that can be designed to degrade the genome of pathogenic bacteria
Discovering new anti-microbial peptides against mycobacteria	Erdogan Gulari	University of Michigan	Design and produce a large library of antimicrobial peptides (AMPS) that will be tested against Mycobacterium tuberculosis strains to identify potential new drugs that can damage the bacterial membrane and be less susceptible to evasion by the development of resistance
Synthetic signals to eliminate essential plasmodium proteins	Andreas Matouschek, Keith Tyo	Northwestern University	Develop synthetic compounds that target essential proteins in the Plasmodium parasite for destruction by its own protein degradation mechanisms
Transcription factor screening for *P. falciparum* therapy	David Segal	University of California, Davis	Develop a high-throughput screen to search for artificial transcription factors (ATF) that are candidates to treat *P. falciparum* infections
*Vaccines*			
A probiotic-based oral synthetic vaccine delivery system	Daniel González	University of Texas at San Antonio	Engineer a probiotic yeast into a strain that can deliver antigens directly to the intestinal mucosal immune system
Adenoviral HIV vaccine vector with CMV-Like immunogenicity	Matt Cottingham	The Jenner Institute, University of Oxford	Engineer an adenovirus vaccine vector that includes HIV antigens as well as the immune evasion genes of cytomegalovirus (CMV)
Bacterial nano-particles as oral vaccines against diarrhea	Garry Blakely	University of Edinburgh	Engineer a common gut bacterium to express antigens from pathogens that cause diarrhea onto nanoscale outer membrane vesicles, as the basis for a new generation of biocompatible oral vaccines
Plant-produced synthetic RNA vaccines	Alison McCormick	Touro University California	Test the ability of a low-cost plant-based synthetic biology method to produce a combined viral protein epitope with an antigen RNA expression system for use in an RNA malaria vaccine
*Biomanufacturing*			
Design of pathways for biofabrication of global health drugs	Linda Broadbelt, Keith Tyo	Northwestern University	Use a computer-aided design (CAD) tool to identify new metabolic mechanisms of action in priority drugs for the developing world, to help optimize methods to produce low-cost versions of these therapeutics in microbes
Development of a microorganism to produce artemisinin	Jay Keasling	Zagaya	Explore the production by an endophytic fungus of artemisinin, a key ingredient in malaria treatments
*Tools and platforms*			
Genetically modified malaria parasites for human challenge	Christian Ockenhouse, Alan Cowman	Walter Reed Army Institute of Research, Walter and Eliza Hall Institute	Generate a transgenic *P. falciparum* malaria parasite that can be used to assess the efficacy of *P. vivax*-based circumsporozoite vaccines
A synthetic biosensor to find drugs targeting TB persistence	Robert Abramovitch	Michigan State University	Use a synthetic biosensor strain and high-throughput screening to discover compounds that inhibit tuberculosis persistence
Reconstitution of a synthetic mycobacterium tuberculosis system	Shaorong Chong	New England Biolabs, Inc.	Synthetically reconstruct essential biological processes of Mycobacterium tuberculosis and use this system as a drug-testing platform for the screening of small-molecule therapeutics against multi-drug resistant *M. tuberculosis*
A predictive model for vaccine testing based on aptamers	Alexander Douglas	Jenner Institute, University of Oxford	Use aptamers to develop a model that can be used to predict the success or failure of new vaccines in clinical trials
Wolbachia as a back door to synthetic entomology	Ichiro Matsumura	Emory University	Use synthetic DNA techniques to transform Wolbachia, a bacterial parasite that infects most insect species, in an effort to engineer mosquitoes to be immune to malaria parasites
A microbial platform for the biosynthesis of new drugs	Christina Smolke	Stanford University	Develop synthetic biology platforms to improve the scale and efficiency of microbial systems used to discover, develop, and produce drugs based on natural products
*Agriculture*			
Engineering plants that make their own fertilizer	Alvin Tamsir, Karsten Temme	Pivot Bio, Inc.	Transfer a nitrogen-fixing gene cluster from naturally occurring bacteria into agricultural crops. The engineered crops could capture and metabolize nitrogen from the atmosphere, reducing the need for petrochemical fertilizers and reducing the cost of farming in developing countries

## Diagnostics/biosensors

A prominent theme was the use of synthetic biology approaches to create novel diagnostics and biosensors, with 13 of the 30 awarded grants falling into this category. A recurring strategy was to engineer whole biological systems—living cells, such as yeast or bacteria, or viruses/phage—to build the molecular apparatus necessary to detect analytes of interest and generate a detectable signal, such as the production of a protein-based pigment. The resulting biosensor could be added to a sample (e.g. blood, urine, water) or, in some cases, could be deployed in vivo in the human body, in the manner of a probiotic. Such biological system-based biosensors have the potential to be relatively low cost to produce and deploy in developing-world environments, leveraging the benefits of biologically replicating systems and pathways. Challenges of the approach (e.g. variation, unpredictability) stem from the inherent complexities of living systems, and include likely difficulties in achieving robust, consistent manufacturing.

Some proposals took the biosensor concept a step further, adding a molecular process intended to kill a specific pathogen downstream of its detection, resulting in a combination diagnostic-therapeutic.

Another theme in the diagnostic category was the use of DNA as a nanomaterial, rather than its more typical role as a diagnostic analyte. The proposed DNA-based sensors would bind specifically to a particular pathogen to signal its presence in a clinical or environmental sample, and have the potential benefit of being highly specific, stable and amenable to field use.

## Therapeutics and vaccines

Many projects proposed to combat a specific infectious pathogen important to global health, either as a therapeutic (i.e., treating an existing infection) or as a vaccine (i.e., preventing infection). Proposed solutions in the former category included novel classes of engineered large molecules, such as synthetic peptides and artificial transcription factors (which might also be useful as tools/reagents for target identification and validation). Other proposed “therapeutics” are actually whole biological systems (cells, viruses), engineered to specifically attack pathogenic organisms. Similarly, numerous projects explored the possibility of genetically engineered whole biological systems, such as commensal yeast, beneficial gut bacteria, or viruses, to produce and deliver antigens for global health pathogens, to be used as an oral/ingested vaccine. Enteric pathogens such as *V. cholerae* and rotavirus were the most popular targets, though some proposals dealt with HIV, TB, and malaria.

Both therapeutics and vaccines based on whole biological systems carry with them the advantages and challenges described above for biosensors. However, the challenges are magnified due to the biosafety concerns and regulatory requirements applicable to an intervention that would be delivered in the human body. Some proposed strategies are so novel that defining and navigating the clinical development and regulatory path is likely to be as significant a potential barrier to eventual deployment in global health as will be the (considerable) technical hurdles.

## Biomanufacturing

A few applications proposed to apply synthetic biology approaches to the problem of producing existing global health interventions, such as drugs against relevant pathogens, typically with the objective of reducing the costs and technical challenges of at-scale manufacturing of critical (and currently limiting) active ingredients (see Vohra and Blakely 2013). Another recurring theme was utilizing biosynthetic pathways to increase chemical diversity, particularly in natural products categories. While biomanufacturing as a general concept is not novel, projects tended to employ a level of systems thinking and tackle a degree of biological complexity that elevates them to the “synthetic biology” category.

## Tools and platforms

There was a strong showing—both in numbers and in creativity—of applications proposing to create tools and platforms with the potential to further research and development in global health. These included designing drug and vaccine discovery systems, such as microbiological systems that expressed human genes for screening inhibitors, or aptamers to accelerate in vivo validation of antigens. Others proposed to build a cell-free synthetic system of a pathogen to screen compounds, using building blocks from both pathogenic and non-pathogenic bacteria. Potential advantages here include discovery tools with more informative and/or more rapid readouts. Another theme was developing and enhancing the synthetic biology toolkit itself, with a focus on enabling work in global health priority pathogens (e.g., *Plasmodium* genetics tools).

## Concluding thoughts

In reviewing the research proposals that were submitted, the question repeatedly arose of what, exactly, constitutes synthetic biology, and whether specific projects and approaches qualified as such. For example, when does a research plan transcend “conventional” genetic engineering and become synthetic biology? Replacing an organism’s entire genome with a synthetic genome seems to clearly fall into the category of synthetic biology, while inserting a single new synthetic gene might not; but what about projects that propose to undertake extensive engineering of a genome, with over a dozen gene insertions and deletions, and even construct novel gene pathways utterly foreign to the host organism? Other proposals seemed conventional at first read, but offered a synthetic biology “twist”; for example, some projects proposed peptide mimetics or biomimicking molecules generated via fairly standard chemical synthesis methodologies, but cast them in roles that required these non-biological components to perform biological functions. See Andrianantoandro et al. (2006) for a review that provides a definition of synthetic biology and distinction versus genetic engineering, along with examples and applications. For our purposes, because the primary objective of the call was to support innovative solutions to global health needs rather than drive the field of synthetic biology per se, in the end we were fairly lenient with the definition of “synthetic biology” in making grant awards.

A few proposals that fell outside of the themes described above gave a glimpse of application areas with large, relatively untapped potential in global health, worthy of consideration when setting future research priorities. For example, the generation of novel enzymes or enzymatic activities, either alone or in concert with more complex metabolic engineering efforts, can be used to design or select for enzymes that perform reactions not found in nature. By generating new enzymatic functions, biological and chemical products of importance to global health could be produced more efficiently and cheaply for use as therapeutics, adjuvants, and a host of other applications, and chemical diversity could be expanded.

Another area with immense promise is the use of synthetic biology to enhance crop nutrition and agriculture. Combining the existing knowledge and tools of genetic engineering in agriculture with emerging synthetic biology approaches has the potential to transform the way agriculture is practiced, yet the majority of progress to date has been in staple crops of importance in developed countries, such as corn and soy. Synthetic biology approaches could play an important role in developing-world agriculture if applied to other crops, including the engineering of novel pathogen resistance, enhanced growth characteristics, or the introduction of exogenous biosynthetic pathways for the production of added nutrition into staple crops. While agricultural applications of synthetic biology were outside of the focus of the GCE grant call, several applications in this category were received, among which one was funded: a proposal to introduce a nitrogen-fixing gene cluster from naturally occurring bacteria into agricultural crops, enabling crops to capture and metabolize nitrogen from the atmosphere.

Given the early stage of the research of the proposed projects and the highly innovative, technically risky strategies employed, as well as the challenges outlined above to delivering effective, affordable global health solutions, it will be years before the impact of the call to “Apply Synthetic Biology to Global Health Challenges” can be measured. However, the initiative can already be declared a success against the objective of drawing the focus of researchers in the space to this area of critical need. Applicants hailed from every inhabited continent, from both public and private institutions, from academia and industry. The high diversity and quality of global health issues, technologies, and strategies in the application pool is an exciting testament to the creativity and capabilities already resident in the synthetic biology research community. There is great opportunity to support continued innovation in this emerging field that can be brought to bear on the needs of the developing world.

## Footnotes

1 See, for example, “Current Uses of Synthetic Biology”, http://www.bio.org/articles/current-uses-synthetic-biology and Peralta-Yahya PP et al., 2012 Aug 16; 488(7411):320–328.

2 Grand Challenges Explorations (GCE) fosters innovation in global health research. The Bill & Melinda Gates Foundation has committed $100 million to encourage scientists worldwide to expand the pipeline of ideas to fight our greatest health challenges. Launched in 2008, more than 850 Grand Challenges Explorations grants have been awarded to innovative, early-stage projects in 50 countries. The grant program is open to anyone from any discipline, from student to tenured professor, and from any organization—colleges and universities, government laboratories, research institutions, non-profit organizations and for-profit companies. The initiative uses an agile, accelerated grant-making process with short two-page applications and no preliminary data required. Initial grants of $100,000 are awarded two times a year. Successful projects have the opportunity to receive a follow-on grant of up to $1 million. For more information about GCE, see http://www.grandchallenges.org/Explorations/.

3 To be considered, proposals were required to closely align with one or more of the Bill & Melinda Gates Foundation’s Global Health priority areas: malaria, HIV, tuberculosis, pneumonia, enteric disease & diarrhea, maternal neonatal & child health, nutrition, polio, and/or family planning. For information about the foundation’s strategies in its priority Global Health areas, see http://www.gatesfoundation.org/global-health/Pages/global-health-strategies.aspx.
